# A Portable Luminometer with a Disposable Electrochemiluminescent Biosensor for Lactate Determination

**DOI:** 10.3390/s91007694

**Published:** 2009-09-28

**Authors:** Antonio Martínez-Olmos, Julio Ballesta-Claver, Alberto J. Palma, Maria del Carmen Valencia-Mirón, Luis Fermin Capitán-Vallvey

**Affiliations:** 1 Ecsens, Department of Electronics and Computer Technology, Granada, Spain; E-Mails: amartinez@ugr.es (A.M.O.); ajpalma@ugr.es (A.J.P.); 2 ECsens, Department of Analytical Chemistry, Campus Fuentenueva, Faculty of Sciences, University of Granada, Spain; E-Mails: juliosci@ugr.es (J.B.C.); cvmiron@ugr.es (M.C.V.M.)

**Keywords:** portable instrument, microcontroller, electrochemiluminescence measurement, disposable biosensor, lactate determination

## Abstract

A hand-held luminometer for measuring electrochemiluminescence (ECL) for lactate determination and based on one-shot biosensors fabricated using screen-printed electrodes is described. The lactate recognition system is based on lactate oxidase and the transduction system consists of electro-oxidation of luminol, with all the reagents immobilized in a Methocel membrane. The membrane composition and reaction conditions have been optimized to obtain adequate sensitivity. The luminometer is based on a large silicon photodiode as detector and includes a programmable potentiostat to initialize the chemical reaction and signal processing circuitry, designed to acquire a low level photocurrent with offset cancelation, low pass filtering for noise attenuation and adjustable gain up to 10^12^ V/A. The one-shot biosensor responds to lactate rapidly, with an acquisition time of 2.5 min, obtaining a linear dependence from 8 × 10^−6^ to 2 × 10^−4^ M, a detection limit of 2.4 × 10^−6^ M and a sensor-to-sensor reproducibility (relative standard deviation, RSD) of around 7–10 % at the medium level of the range.

## 1. Introduction

L(+)-Lactate is produced in the anaerobic metabolism of glucose and its determination is of interest in clinical analysis, sports medicine and food analysis. The measurement of lactate is routinely performed with liquid chromatography, spectrophotometry and amperometry, mainly with enzymatic electrodes [1-3]. In the foodstuffs field, lactate is produced by bacterial fermentation and is an essential component related to the manufacture of cheese, yoghurt, milk, and so on, making the monitoring of lactate an important quality control parameter.

Rapid evaluation of lactate levels can be performed with one-shot sensors that have been developed over the last two decades, mostly amperometric ones [4], based on the change in concentration of a redox-active reactant or product via an analyte specific enzyme reaction. Different enzyme biosensors for lactate have been described, mainly, cytochrome b2, lactate monooxygenase and lactate oxidase immobilized on different types of polymers [5], conducting polymers [6] or nanomaterials [7]. Different types of screen-printed amperometric disposable sensors have been described for lactate and some of them are on the market, especially the fitness market [8].

Chemiluminescence measurement is a useful and versatile technique with typically low detection limits, and has been used for sensing lactate based on enzymatic systems, chiefly lactate oxidase/peroxidase/luminol [9] and lactate dehydrogenase/NADH/bioluminescent enzymes [10] implemented in different types of flow [11,12] or fiber optic formats [9].

Nevertheless, chemiluminescent detection has rarely been used in disposable sensors despite its advantages of sensitivity and simple instrumentation, because of the need to immobilize all the chemistries required and the time control to trigger the sensing reactions upon contact with the sample. The use of electrochemiluminescence (ECL) can offer clear advantages for controlling the chemical system [13,14]. In this context, the use of screen printing technologies, with their benefits of low cost and mass production, appears to be interesting for developing ECL one-shot biosensors along with portable instrumentation.

Different techniques have been developed to detect the light resulting from luminescence and electroluminescence reactions. Those processes are commonly controlled by a potentiostat or an amperometric unit [15-17], which establishes potential differences or electric current flow between the electrodes of the cell, respectively. When there are no requirements regarding the volume or the power supply needed for the design, a good solution to detect the low light intensity generated by the CL reaction is the use of a photomultiplier tube (PMT) [18,19].

These devices produce a great amount of electrons when a photocathode is exposed to an ultra low photon flux. These photodetectors need a very high voltage supply, up to thousands of volts. This fact, together with its bulky size, makes the PMT an inconvenient device for the development of portable instrumentation. Another technique for the detection of luminescence that can be found in the literature consists of the use of CCD devices, such as CCD cameras and detectors [20,21]. These devices have the main disadvantage that in order to achieve good resolution; they need a working temperature of tens of Celsius degrees below 0 °C. Moreover, using and implementing them is complex.

The use of organic and semiconductor photodiodes such as photodetectors for the registration of CL radiation is also well discussed in the literature [12,22], for example, the generic miniature complementary metal oxide semiconductor (CMOS) photosensing chip for the determination of glucose developed by Chiun Jie-Yuan *et al.* [23]. One generic microfluidic device has even been designed with both types of detectors: PMT and silicon photodiodes in printed circuit board (PCB) technology [24]. Photodiodes are small devices and are thus easily integrated into a measurement system. The sensitivity of these photodetectors depends on the inverse polarization applied to them, which can vary from a few volts to hundreds of volts, in the case of avalanche photodiodes. This voltage supply can be generated from battery arrangements, by means of a simple DC-DC boost converter. Therefore, photodiodes are an appropriate solution for the development of portable instrumentation to measure luminescence from ECL reactions, which is one of the goals of this work.

The portable instrumentation developed here was applied to lactate determination using a biosensor based on the atmospheric oxidation of lactate catalyzed by lactate oxidase, producing hydrogen peroxide that reacts with electrochemically oxidized luminol in basic medium, producing the ECL emission. The main advantages of our design lie in its portability, low cost because of the use of a photodiode instead of a costly or bulky photomultiplier, and the use of only a few microliters of sample analysis.

## 2. Experimental Section

### 2.1. Chemicals and Materials

L-(+)-Lactate 10^−2^ M stock solutions in pH 9.0 phosphate buffer 0.2 M were prepared using 97% L‐(+)-lactate lithium salt. Solutions of lower concentrations were prepared by dilution as appropriate. Buffers, made with Tris 0.5 M (Trizma base 99%) and phosphate 0.5 M (Na_2_HPO_4_) and adjusted to different pH by adding NaOH or HCl, were prepared. All of these compounds were supplied by Sigma (Sigma-Aldrich Química S.A., Madrid, Spain). Other reagents were: 10^−2^ M luminol (5-amino-2,3-dihydro-1,4-phthalazinedione) stock solution from 97% luminol, Methocel^©^ 90HG (hydroxypropyl-methyl cellulose), 96% bovine serum albumin (BSA) powder, and lactate oxidase (LOx) from *Pediococcus spp.*, 39 IU/mg prepared in pH 7.0 phosphate buffer 0.1 M; all reagents were supplied by Sigma. Reverse-osmosis type quality water (Milli-Q Plus185 from Millipore, Molsheim, France) was used throughout.

Screen-printed electrochemical cells were supplied by Palmsens Instruments BV (Ruitherland, The Netherlands) and Dropsens (Oviedo, Spain). They consist of a round-shaped graphite working electrode, a graphite counter electrode and a silver pseudo-reference electrode on a plastic or ceramic support. Before being used, the electrochemical cells were tested for uniform behavior. In order to prepare a receptacle on the disposable electrochemical cell, the electrode area was covered with successive layers of plastic white adhesive tape up to 1 mm thick with an 8 mm diameter hole (50 μL volume) in the sensing area.

### 2.2. Apparatus and Software

For comparison purposes, the ECL emission from the screen-printed disposable biosensor cells was measured using a H8529 photomultiplier (PMT) interfaced by a C8855 USB photo counting unit, both from Hamamatsu Technologies, connected to a personal computer. The potentiostat used was a PS-PC1 model Palmsens from Ivium Technologies (Eindhoven, The Netherlands) with a connector for the screen-printed electrodes. The arrangement used for ECL emission has been described elsewhere [25]. Other instruments consist of a Crison digital pH-meter (Crison Instruments, Barcelona, Spain) with combined glass-saturated calomel electrode and a sourcemeter instrument 2636A from Keithley used for current-voltage converter characterization. Software programs used were: Statgraphics software package (Manugistics Inc. and Statistical Graphics Corporation, USA), ver. 5.0 (2000) for data treatment; Microsoft Office 2003 and Visual Basic 6.0.

### 2.3. Disposable Biosensor Preparation

The lactate sensitive membranes were made using a solution prepared in a 3 mL glass vial with 0.51 mg of luminol (2.9 × 10^−3^ M), 2.5 mg of BSA (100 g/L), 12.5 mg of Methocel (49.9 mg/mL), 50 μL of 12.5 IU/mL LOx and 200 μL of pH 8.5 phosphate buffer (0.2 M). The solution was shaken and ultrasonicated for 10 min and then stored in a refrigerator at 4 °C. The disposable biosensors were cast by placing 5 μL of the prepared solution on the working electrode of the electrochemical cell. After this, the biosensors were dried at room temperature for 1 h and then stored in a refrigerator at 4 °C until use.

### 2.4. Procedure for Lactate Determination

Aqueous standard solutions of lactate or samples containing between 10^−7^ and 10^−3^ M in working buffer, consisting of pH 9.0 phosphate buffer 0.2 M containing 0.25 M of NaCl, were prepared. A volume of 35 μL of standard or sample solution were taken and deposited in the receptacle of the screen-printed cell. The lactate sensitive biosensor was placed in the electrical socket of the portable instrument and covered with the lid that held the photodiode. The instrument was configured to wait for two minutes after connection, in order to allow time for the enzymatic lactate conversion, applying then three pulses of 1 s with 10 s between them at 0.5 V. The resulting ECL emission that occurred was collected by the photodiode without wavelength discrimination. The analytical signal and calibration are described below.

### 2.5. Measurement Electronics Description

In this section, a hand-held luminometer is presented. This instrument is a simple and portable tool able to provide, within a brief period of time, the concentration of lactate with a lower detection limit than previous portable lactate-meters.

#### Built-in Potentiostat

The core of the signal acquisition consists of a solid-state photodiode detector, which generates an electric current proportional to the ECL being measured. Silicon photodiodes have proven to be useful for the study of chemiluminescence reactions [26,27]. This whole system is designed to detect very weak light emissions.

**Figure 1.**
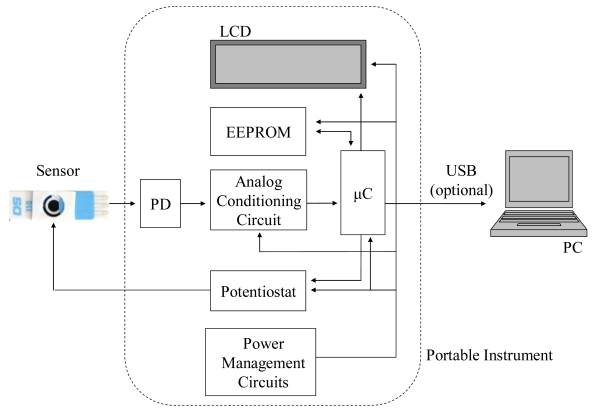
Block diagram of the instrument.

The functional block diagram of the proposed electronic system is detailed in Figure 1. The biosensor, which consists of an electrochemical cell containing the problem substance, is placed directly under the photodiode (PD). The ECL reaction takes place on the cell surface when a voltage difference is applied between the working and the reference electrodes in the biosensor. This polarization of the sensor is carried out using a programmable built-in potentiostat, which is designed to apply variable voltage steps between the sensor electrodes. The potentiostat system, formed by the potentiostat circuit and the electrochemical cell, accurately maintains the voltage between the electrodes during the measurement time [28].

**Figure 2.**
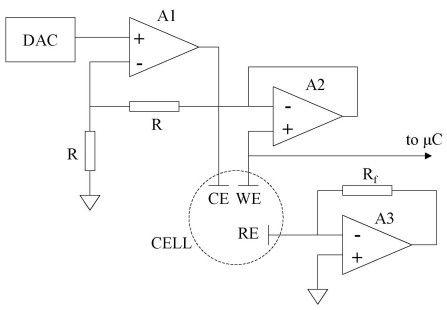
Potentiostat and drop monitoring system.

The design of the potentiostat used in the proposed luminometer is presented in Figure 2. In this circuit, the digital-to-analog converter (DAC) generates an analog voltage from a 16-bit digital word sent by the microcontroller, which is the input value to the potentiostat. If the electrochemical cell is full of a conductive liquid, the operational amplifiers A1 and A2 form a non-inverting amplifier stage with gain 2. This establishes a voltage at the working electrode that is double the input voltage value. The voltage at the reference electrode is forced to virtual ground because of the negative feedback of the operational amplifier A3. Thus, the voltage difference between the working and the reference electrodes is simply twice the analog value generated by the DAC. This architecture allows the user to apply voltage steps between the electrodes with a resolution of 8 mV.

The voltage at the working electrode is monitored directly by the microcontroller. The potential at this electrode changes with the presence of the test drop on the electrochemical cell surface thus can be used to detect the precise instant when the drop is deposited on the biosensor. This event causes the start of a time count, thus allowing a precise determination of the time elapsed between the drop deposit and the beginning of the measurements. Therefore, the timing control of the measurement procedure can be achieved with this potentiostat.

#### Photocurrent conditioning circuit

Several photodiodes were tested, in order to maximize the response to light excitation, such as the planar-shaped S1227-66BR, S1227-33BR, S1226-44BK and S1226-8B5, all from Hamamatsu Photonics, with the main difference among them being the diode area; or the non-planar-shaped models OSD5-5T and OSD15-5T (Centronic, Croydon, UK). Finally, model S1227-66BR (Hamamatsu Photonics, Shizouka, Japan) was selected for this work, due to its high sensitivity, low dark current, and broad active area (33 mm^2^), which fits the size of the electrochemical cell, as well as its planar geometry, which allows for a very close arrangement of the structure composed of the photodiode and the ECL screen-printed cell.

**Figure 3.**
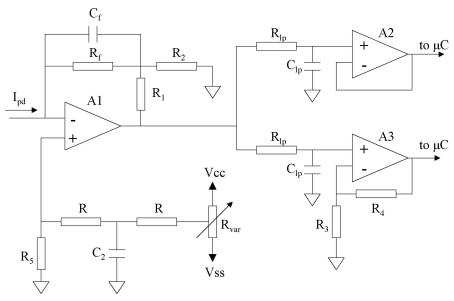
Measurement circuit.

The measurement of the current generated by the photodiode in response to the ECL emission is carried out by means of a very high gain current-to-voltage converter, shown in Figure 3. First, the current is converted into voltage through the stage formed by the operational amplifier A1 (TLC277, Texas Instruments, Dallas, USA). This device has a feedback T-network composed of the resistors *R_f_, R_1_* and *R_2_*, which can result in a high gain conversion, with the output voltage of A1:

(1) $$V_{o}=\frac{V^{+}(R_{1}+R_{2})-I_{pd}[R_{1}(R_{2}+R_{f})+R_{f}R_{2}]}{R_{2}}$$

where *V*^+^ is the voltage at the non-inverting input of the operational amplifier A1, and *I_pd_* is the current generated by the photodiode. As has been measured, the voltage *V*^+^ takes a value of a few mV which can be modified through the variable resistor *R_var_*. The effect of this resistor is to nullify the input offset voltage of the operational amplifier, which has a high influence on the output because of the T- network topology and the large gain of this stage [29].

Under ideal conditions (*V*^+^ = 0) obtained varying *R_var_*, and assuming *R_f_* ≫ *R_2_*, Equation (1) can be rewritten as:

(2) $$V_{o}=-I_{\text{pd}}R_{f}\left(1+\frac{R_{1}}{R_{2}}\right)$$

Selecting a high value for *R_f_* and making *R_1_*>*R_2_*, a gain factor of 10^9^ – 10^11^ V/A can be achieved. Equation (2) provides a linear relation between the current from the photodiode and potential output of A1.

The output voltage of A1 was further conditioned using two parallel stages, formed by the operational amplifiers A2 and A3. In each stage, the signal was first filtered through an *RC* low-pass filter with cut frequency of 10 Hz for noise attenuation. The operational amplifier A2 acts as a buffer, whereas A3 amplifies the output voltage of I/V converter before sending it to the microcontroller. In this way, the μC receives two signals, one corresponding to the filtered output of the first stage, and another that is an amplification of the latter one. The purpose of having two different channels for measuring the same signal is to expand the range of lactate that can be analysed. The output signal of A2 is selected when the lactate concentration is high, since it will lead to higher pulses of the output voltage of the converter stage A1. These high voltage pulses may cause the output of the amplifier stage A3 to reach the value of the supply voltage, thus being distorted by this limit. On the other hand, if the lactate concentration in the test drop is low, the light produced in the ECL reaction is also low, as is the output signal of A1. This signal will need to be amplified, thus selecting the output of the stage A3 as the input channel to the microcontroller. The outputs of A2 and A3 are connected directly to the microcontroller (μC) (PIC18F2550, Microchip Inc., Chandler, USA), which uses an internal 10-bit analog-to-digital converter to alternatively sample these signals at high frequency. A serial EEPROM module (24LC512, Microchip Inc) of 512 kbits is used to store the sampled data. Finally, once the calibration function (see next section) programmed in the microcontroller is applied, the results are sent to the LCD display (Figure 1).

The instrument includes electronic circuitry for power management from 2 standard 1.2 V AAA batteries or from a standard AC-DC adapter. This circuitry is based on the use of the DC-DC boost converter LM2623 (National Instruments, Austin, TX, USA), which provides an adjustable output voltage of 1.4 to 14 V. In this case, an output of 5 V for both the analog and the digital circuitry of the instrument was selected. All electronic circuitry is included in an enclosure with optical, magnetic and electrical shielding with external dimensions 75 × 150 × 25 mm and 212.5 g weight. Moreover, control software written in Visual Basic allows the user to optionally communicate the instrument with a computer via a USB port to receive the data for further analysis.

## 3. Results and Discussion

### 3.1. Lactate Biosensor

**Figure 4.**
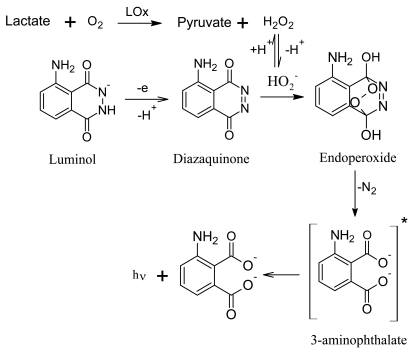
ECL emission mechanism of luminol when lactate and the enzyme (LOx) are in the screen-printed cells.

Lactate determination is based on its atmospheric oxidation catalyzed by LOx producing hydrogen peroxide, which reacts with electrochemically oxidized luminol in a basic medium, generating an ECL emission at 425 nm (see Figure 4). For this purpose, we need to immobilize all of the reagents needed to make the lactate recognition and transduction possible. To immobilize LOx and luminol in the working electrode of screen-printed cells, Methocel polymer was selected, because these membranes offer the best adherence, good electrical properties and a hydrophilic character as was discussed in a previous work [25].

If the medium is more alkaline, a higher emission is obtained, due to an increase in luminol deprotonation [30-32]. In the absence of lactate, a background ECL signal is observed that starts to grow from pH 9.0 up to 10.5 (the background ECL signal increases 206.3 V/s per pH unit). This background signal is due to the reaction of the dissolved oxygen present in the solution with the oxidized luminol and also to the more efficient production of excited 3-aminophthalate with pH [33]. Thus, the quantification of lactate must be performed in weakly alkaline conditions because the increases in the background produce lower precision. This background signal is observed using conventional detectors such as photomultipliers or CCD cameras but not with the developed portable photodiode luminometer, due to offset cancelation before each measurement, as explained above. With respect to temperature conditions, we observed that the enzymatic chemical reaction and the luminescence emission is nearly the same in the 20–25 °C temperature range.

For the preparation of the biosensor, we optimized the chemical conditions for the different components needed: luminol (2.9 × 10^−3^ M), LOx (12.5 IU/mL), BSA (100 g/L), and electrolyte (pH 8.5 phosphate buffer 0.2 M). All these reagents were immobilized by entrapment with a Methocel cellulose membrane (49.9 mg/mL) placed on the working electrode of a screen-printed commercial graphite electrochemical cell [25].

### 3.2. I/V Converter Calibration

The I/V converter shown in Figure 3 was calibrated and tested using several combinations of the resistances *R_1_, R_2_* and *R_f_*, since a high value of the conversion factor is required. Combinations such as *R_f_* = 10 MΩ, *R_1_* = 100 kΩ, *R_2_* = 1kΩ and *R_f_* = 100 MΩ, *R_1_* = 100 kΩ, *R_2_* = 1 kΩ result in gain factors of 10^9^ and 10^10^ V/A, respectively, and a linear range of 50 to 4000 pA and 10 to 400 pA. From these results, it was concluded that an increment of the conversion factors results in a decrease of the input range in which the response of the I-V converter is linear. Given that the light generated in this ECL process results in a current from the selected photodiode in low intensity, below 50 pA, the values of the resistances that provide a linear range matching this low intensity are those cited above, *R_f_* = 100 MΩ, *R_1_* = 10 MΩ, *R_2_* = 1 kΩ, which results in a gain of 10^11^ V/A.

**Figure 5.**
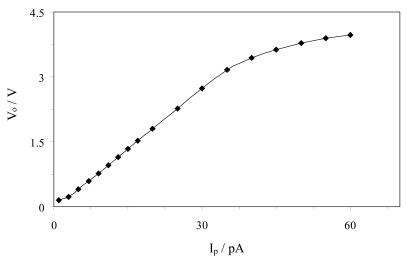
Calibration of the current-to-voltage converter.

A calibration of the converter stage is presented in Figure 5. This calibration was carried out using the sourcemeter instrument 2636A (Keithley, Cleveland, USA), for the values of the resistors *R_f_* = 100 MΩ, *R_1_* = 1MΩ and *R_2_* = 1kΩ. As can be seen, this circuit has a linear response in the range of 3 to 35 pA. These limits are properly set to detect emissions of very low intensity light, like those resulting from the ECL reaction on this biosensor, as will be shown below.

### 3.3. Measurement Conditions and ECL Analytical Signal

The emission of ECL by the luminol reaction occurs in alkaline medium and when positive potentials are applied [14,34]. Working in chronoamperometric mode with the two screen-printed graphite cells described above (Florence and Dropsens), the ECL signal starts at 0.3 V potential [35] and the intensity increases steeply as the potential increases, reaching a maximum for 0.5 V. For that reason, we selected this potential for working conditions.

The main apparent difference between the two types of commercial screen-printed graphite cells tested is related to the working electrode diameter (3 mm and 4 mm, respectively) and the material support: plastic and ceramic. The size of the working electrode seems to be responsible for the enhanced sensitivity, some fourfold, of Dropsens cells–which were selected–over the Florence cells.

We studied two possible analytical parameters working in chronoamperometric mode: ECL intensity using only one short pulse (<4 s) or an initial rate. The second parameter offers the best response and better detection limits for lactate (3.5 × 10^−5^ M for short pulse and 2.4 × 10^−6^ M for initial rate), and for that reason was used for the rest of the study.

**Figure 6.**
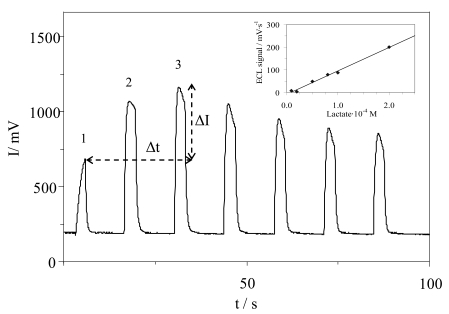
Luminol ECL emission at lactate 10^−4^ M in chronoamperometric mode (0.5 V). Lactate linear calibration is presented in the inset.

The ECL signal varies over time due to enzymatically catalyzed hydrogen peroxide generation. For a constant amount of lactate, the ECL transient signal coming from successive potential pulses of the same voltage grows with time, reaching a maximum in the third ECL peak and then decreasing, due to the enzymatic kinetic and luminol consumption (Figure 6).

The initial rate for different lactate concentrations obeys a fast Michaelis-Menten kinetic. This rate can be estimated more easily using a two point approximation, as shown in Figure 6. In this way, it is not necessary to obtain the initial reaction rate from the ECL profiles measured using multiple pulses of the same potential at short times, but using the difference in intensities (_ΔIECL_) at two pulses spaced in time _Δt_, which give us an estimation of rate $\left({\text{v}}_{\text{ECL}}=\frac{\Delta {\text{I}}_{\text{ECL}}}{\Delta \text{t}}\right)$, in mV/s units. Since a background ECL emission does not exist, it was not necessary to correct the analytical signal v_ECL_ from the blank.

**Figure 7.**
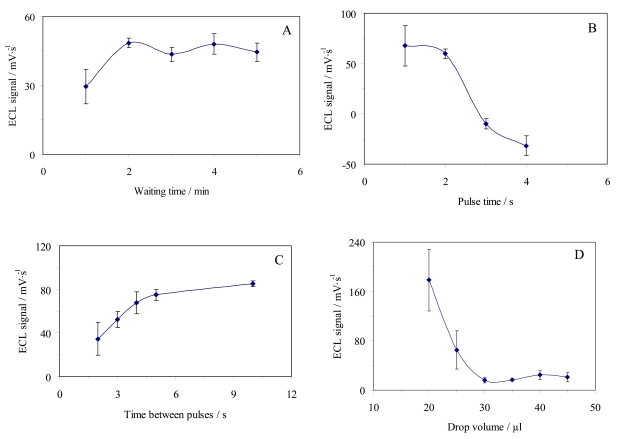
Electrical variables for ECL lactate determination: (A) waiting time; (B) pulse time; (C) time between pulses; and (D) drop volume.

We then studied the different experimental conditions for the measurement of the analytical parameter selected: (A) waiting time before measurement, (B) pulse time, (C) time between pulses, and (D) sample volume (Figure 7).

- The analytical signal increases when the waiting time increases up to 2 min, remaining constant onwards (Figure 7A). This could be attributed to the limiting effect of H_2_O_2_ at low waiting times. At higher waiting times, the signal is constant due to the compensation by the higher H_2_O_2_ concentration and the limiting effect of luminol present. 3 min was selected as the waiting time to ensure the independence of this factor.
- The higher pulse time the lower signal was, as shown in Figure 7B, due to the increase in H_2_O_2_ consumption with the time pulse, which results in a lower ECL increasing (ΔI_ECL_) between pulses. Thus we chose 1 s as the pulse time to avoid the observed decrease. (C) On the other hand, the use of a higher time between pulses means an increase in H_2_O_2_ production and hence in the signal because of the enzymatic kinetic of this biosensor (Figure 7C). Then, 10 s was chosen as optimal because the ECL signal becomes stable at values of more than 5 s.
- The sample volume placed in the receptacle of the biosensor was studied, showing that the ECL signal decreases one order of magnitude on average when the volume increases from 20 to 30 μL, then increasing slowly for higher volumes up to 45 μL, the maximum capacity of the receptacle (Figure 7D). Volumes of less than 25 μL do not cover the whole cell and the applied potential can experience some fluctuations that result in the apparently higher signals along with high irreproducibility. Consequently, we selected 35 μL as the sample volume because of its good precision (7.2% RSD).

In order to obtain the best analytical characteristics, we studied the way to acquire the analytical parameter v_ECL_ $\left({\text{v}}_{\text{ECL}}=\frac{\Delta {\text{I}}_{\text{ECL}}}{\Delta \text{t}}\right)$ from the intensity values of the initial pulses. Table 1 shows the analytical characterization using the analytical signal coming from the 1^st^, 2^nd^ and 3^rd^ pulse (see Figure 6), namely v_ECL1-2_, v_ECL1-3_ and v_ECL2-3_. Comparing the experimental results, we observed the best linearity, sensitivity and detection limits for v_ECL1-3_ parameter and similar relative standard deviations (RSD) for a 2 × 10^−4^ M lactate concentration.

The ECL signal increases when the distance between the screen-printed electrode and the photodiode decreases due to the improved collection of photons at short distances. For that reason, a distance of 2 mm was selected instead of 1 mm because the shorter distance (1 mm) increases the scattering of radiation by some drop lens effect and soaking of the photodiode can occur.

### 3.4. Calibration

The dependence of the ECL signal with the lactate concentration was studied between 10^−7^ and 10^−3^ M, obtaining a linear relationship from 8 × 10^−6^ to 2 × 10^−4^ M (see inset in Figure 6). From the point of view of signal processing, as noted in the instrument description, after the current-to-voltage converter, there are two voltage conditioning circuits in parallel to increase the sensitivity at low signals. Both include a low pass filter for noise and interference attenuation, one with no additional gain and the second amplifier with a gain of 10.0 V/V. Therefore, accounting for the 10^11^ A/V converter gain, the total maximum gain is 10^12^ A/V with a −3 dB cut frequency at 10 Hz. This maximum gain is used for low lactate concentration, below 10^−5^ M, whereas the gain of 10^11^ A/V is sufficient to measure above that point. The analytical function was obtained by means of a calibration set composed of five standards with five replicates each, showing the pattern: v_ECL1-3_ = a·[lactate] + b. In this case and with an instrument gain of 10^11^ A/V, a sensitivity of 195.4 V·s^−1^·M^−1^ was obtained. Using the standard criteria [36], the limit of detection (LOD) calculated was 2.4 × 10^−6^ M. The repeatability of biosensor, expressed by RSD, was defined from five different prepared biosensors at different lactate concentrations obtaining values ranging from 7 to 10%.

### 3.5. Technical Specifications and the Lifetime of the Biosensor

The sensitivity of the detection depends on the photodetector used. There are four most common photodetectors: the photomultiplier, the photodiode, the avalanche photodiode and the CCD array. The features of the measurement determine which detector it requires. In fact, one generic application might use different detector types depending on the specific implementation. In our case, with diffuse light coming from an ECL reaction, a detector with a large area is required. Taking into account cost, photodetectors are a good choice that also avoids complicated electronics to bias an avalanche photodiode or to process the output of CCD arrays. As will be shown below, our instrument based on a silicon photodiode presents comparable specifications to other more sophisticated solutions based on photomultiplier and CCD cameras.

This study is presented in Table 2. Similar specifications from the photodiode can be seen with respect to the photomultiplier in linear range, limit of detection and linearity, but in the case of reproducibility the photodiode has a higher value than the PMT and CCD camera (RSD of 2 × 10^−4^ M lactate value). With respect to the CCD camera, the dynamic linear range and detection limits are better than the photodiode detection.

The explanation for the higher RSD obtained using the photodiode when compared to the results obtained with PMT (see Table 2) is attributed to misalignment effects and the variation in the distance from electrochemical cell to photodiode. A minimal position variation of the screen-printed cell (some tenths of millimetres) can generate different ECL signals due to photodiode sensibility with source light distance.

The lifetime of the disposable biosensor was studied protected from light and preserved at 4 °C using a series of prepared biosensors and regularly checking their response with the portable luminometer described at a lactate concentration of 10^−4^ M. The lifetime obtained was around two months. The cost of the biosensor, considering reagents, screen-printed cells and support for membrane making is around 1.26 €/unit.

## 4. Conclusions

A new hand-held luminometer for an electrochemiluminescence (ECL)-based one-shot biosensor for lactate is described. This instrument represents a breakthrough because of its very low detection limit and, because until now, it was only possible to obtain a compact measurement system for lactate determination with an electrochemical sensor. The use of a solid-state photodiode as optical detector, instead of a photomultiplier, which is the usual technique in bulky commercial ECL systems available now, as well as the integration of the potentiostat and the measurement electronics in the same printed circuit board, results in a low-cost and compact instrument. The microcontroller makes accurate synchronization and timing of the chemical reaction possible as well the control of the precise signal processing electronics. Given the very low light intensity, circuitry for offset cancelation and gain selection has been included.

Exciting a sample volume of 35 μL with only three consecutive steps of 0.5 V and measuring the light resulting from the reaction on the sensor with an analysis time of 2.5 min provides a method for the evaluation of lactate concentration. The possibility of using screen-printed graphite electrodes leads to portable and simple instrumentation. The incorporation of a drop detector in the instrument makes automatic lactate measurement possible, without the need for trained personnel. The result of the lactate concentration can be easily read on the LCD screen or on a PC using a USB cable. A good linear calibration in the range of 8 × 10^−6^ to 2 × 10^−4^ M was achieved, which indicates that lactate in biological and food samples can be measured.

## Figures and Tables

**Table 1.** Analytical parameters for different initial rate estimation.

**Parameter**	**Initial rate estimation**		

	**v_ECL1-2_**	**v_ECL1-3_**	**v_ECL2-3_**

Linear range / (M)	2 × 10^−5^ – 2 × 10^−4^	8 × 10^−6^ – 2 × 10^−4^	8 × 10^−6^ – 2 × 10^−4^
Intercept (b) / (V/s)	-0.5	0.5	-2.5
Slope (a) / (V·s^−1^·M^−1^)	155.8	195.4	137.6
r^2^	0.938	0.998	0.925
Detection limit / (M)	3.2 × 10^−6^	2.4 × 10^−6^	1.8 × 10^−5^
RSD lactate (%) 2 × 10^−4^ M	11.1%	10.3%	11.0%

**Table 2.** Comparison of different detection instruments for lactate ECL on screen-printed graphite cells.

**Parameter**	**Photodiode (This work)**	**PMT [25]**	**CCD camera [34]**
Linear equation	v_ECL_ = a· [lactate] + b	log v_ECL_ = a·log[lactate] + b	I_ECL_ = a· [lactate] + b
Linear range / (M)	8 × 10^−6^ – 2 × 10^−4^	10^−5^ – 5 × 10^−4^	3 × 10^−7^ – 10^−4^
r^2^	0.998	0.996	0.972
Detection limit / (M)	2.4 × 10^−6^	5.0 × 10^−6^	3.0 × 10^−7^
RSD lactate (%) 2 × 10^−4^ M	10.3%	3.3%	4.4%
